# Effects of obesity on insulin: insulin-like growth factor 1 hybrid receptor expression and Akt phosphorylation in conduit and resistance arteries

**DOI:** 10.1177/1479164118802550

**Published:** 2018-10-08

**Authors:** Romana S Mughal, Katherine Bridge, Irma Buza, Rita Slaaby, Jesper Worm, Gro Klitgaard-Povlsen, Henning Hvid, Marianne Schiødt, Richard Cubbon, Nadira Yuldasheva, Anna Skromna, Natallia Makava, Grith Skytte-Olsen, Mark T Kearney

**Affiliations:** 1 Division of Cardiovascular and Diabetes Research, Leeds Institute of Cardiovascular and Metabolic Medicine (LICAMM), School of Medicine, University of Leeds, Leeds, UK; 2 Global Research, Novo Nordisk A/S, Malov, Denmark

**Keywords:** Obesity, IGF-1 receptors, hybrid receptors

## Abstract

Insulin and insulin-like growth factor-1 stimulate specific responses in arteries, which may be disrupted by diet-induced obesity. We examined (1) temporal effects of high-fat diet compared to low-fat diet in mice on insulin receptor, insulin-like growth factor-1 receptor, insulin receptor/insulin-like growth factor-1 receptor hybrid receptor expression and insulin/insulin-like growth factor-1-mediated Akt phosphorylation in aorta; and (2) effects of high-fat diet on insulin and insulin-like growth factor-1-mediated Akt phosphorylation and vascular tone in resistance arteries. Medium-term high-fat diet (5 weeks) decreased insulin-like growth factor-1 receptor expression and increased hybrid expression (~30%) only. After long-term (16 weeks) high-fat diet, insulin receptor expression was reduced by ~30%, insulin-like growth factor-1 receptor expression decreased a further ~40% and hybrid expression increased a further ~60%. Independent correlates of hybrid receptor expression were high-fat diet, duration of high-fat diet and plasma insulin-like growth factor-1 (all *p* < 0.05). In aorta, insulin was a more potent activator of Akt than insulin-like growth factor-1, whereas in resistance arteries, insulin-like growth factor-1 was more potent than insulin. High-fat diet blunted insulin-mediated vasorelaxation (*p* < 0.01) but had no effect on insulin-like growth factor-1-mediated vasorelaxation in resistance arteries. Our findings support the possibility that hybrid receptor level is influenced by nutritional and metabolic cues. Moreover, vessel-dependent effects of insulin and insulin-like growth factor-1 on vascular tone and Akt activation may have implications in treating obesity-related vascular disease.

## Introduction

Acting via their cognate receptors insulin and insulin-like growth factor-1 (IGF-1) respond to environmental cues and nutrient availability to coordinate metabolism and growth.^1^ To do this, insulin and IGF-1 may act on multiple tissues, including the vascular endothelium where they activate endothelial nitric oxide synthase (eNOS) activation of the upstream kinase Akt.^2^ In aorta, we have shown that insulin and IGF-1 stimulated vasorelaxation and activation of eNOS is blunted in obesity.^2^

The insulin receptor (IR) and IGF-1 receptor (IGF-1R) are heterodimers consisting of two extracellular α-subunits and two transmembrane spanning β-subunits held together by disulphide bonds.^3^ Homology between IR and IGF-1R is high and as a result they can heterodimerise to form hybrid receptors composed of one IGF-1Rαβ complex and one IRαβ subunit complex.^4^ The proportion of hybrid dimerisation is thought to be a function of the molar fraction of each receptor in the ER.^5^ According to this model, a marked increase in IR leads hybrids to form in preference to IGF-1R homodimers.^6^ Hybrid receptors are thought to have a binding affinity similar to the IGF-1R, that is, binding IGF-1, but not insulin, with high affinity.^7^ By reducing IR availability, the formation of hybrid receptors has been suggested to have a negative regulatory effect on insulin signalling.^8,9^

In cross-sectional studies in humans with insulin resistance of relatively short duration, increased hybrid receptor expression is not seen,^10^ whereas patients with type 2 diabetes have down-regulation of IR and increased expression of hybrids.^11^ The temporal relationship between expression of IR, IGF-1R and hybrids in obesity and their pathological correlates in the vasculature in vivo remains unclear. Moreover, whether IGF-1 has vasorelaxant effects in resistance arteries and the effect of obesity on these responses is also unclear. To answer these questions we examined (1) the temporal changes in expression of IGF-1R, IR and hybrids in aorta and their correlates in high-fat diet-induced obesity; (2) the effect of different pathological insults associated with obesity on IGF-1R, IR and hybrid expression in human endothelial cells; and (3) the effect of IGF-1 and insulin on resistance vessel tone and Akt phosphorylation and the influence of obesity on these responses.

## Methods

### Animals and animal procedures

C57BL/6J male mice were purchased from Jackson Laboratories and acclimatised for 7 days before starting experimental procedures. Mice were maintained in a temperature and humidity-controlled environment on a 12-h light:dark cycle. Male mice were studied in all experiments which were conducted in accordance with accepted standards of humane animal care under UK Home Office Project licence No. P144DD0D6.

### Diet-induced obesity

Mice were rendered obese by placing them on a 60% high-fat diet (HF; diet D12492, Research Diets Inc. New Brunswick, NJ, USA). Age-matched littermate controls were placed on a 10% low-fat diet (LF; diet D12450B, Research Diets Inc. New Brunswick, NJ, USA). All mice were fed standard chow [Special Diet Services, CRM P(PB), Dietex International] until reaching 6 weeks of age, at which point diets were switched to either HF or LF for 2, 5 or 16 weeks.

### In vivo examination of glucose homeostasis

In vivo metabolic testing was performed as previously described;^12,13^ for glucose tolerance tests (GTT), mice were fasted for 6 h, followed by intraperitoneal (IP) injection of 2 mg/kg glucose after which blood glucose was determined at 30-min intervals by tail vein sampling using a portable glucometer (Accu-chek Aviva; Roche Diagnostics, Burgess Hill, UK). For analyses of plasma insulin and IGF-1, blood was sampled at euthanasia from the inferior vena cava. Blood was sampled at euthanasia from the inferior vena cava. Plasma insulin and IGF-1 were measured using ultrasensitive mouse enzyme-linked immunosorbent assay (ELISA) kits (CrystalChem, Downers Grove, IL and R&D Systems, Bio-Techne, MN) as previously described.^12^

### Quantification of IRs and IGF-1Rs

Mice were euthanised at 2, 5 or 16 weeks after feeding and aortae harvested and snap-frozen. Tissue was processed for analysis by Western blotting to examine changes in receptor protein expression. Samples were mechanically lysed in cell extraction buffer (Invitrogen, Carlsbad, CA, USA) with inhibitors, using a TissueLyser (QIAGEN, Dusseldorf, Germany). Protein was quantified by the bicinchoninic acid assay (BCA) (Sigma-Aldrich, St. Louis, MO, USA). Twenty micrograms of protein was resolved on a 4%–12% Bis–Tris gel (Bio-Rad, Hertfordshire, UK) and transferred to nitrocellulose membranes. Membranes were probed with antibodies diluted in 5% bovine serum albumin (BSA); 1:1000 insulin receptor-beta (clone 4b8), 1:1000 IGF-1 receptor-beta (clone D23H3) and 1:20,000 beta actin (Cell Signaling, MA, USA), before incubation with appropriate secondary horseradish peroxidase (HRP)-conjugated antibody (Dako, Glostrup, Denmark). All antibodies are summarised in Table 1. Blots were visualised with Immobilon Western Chemiluminescence HRP Substrate (Merck Millipore, Hertfordshire, UK) and imaged with Syngene chemiluminescence imaging system (SynGene, Cambridge, UK).

**Table 1.** Antibody details.

Peptide/protein target	Name of antibody	Manufacturer catalogue #	Species raised, monoclonal or polyclonal	Dilution used
IR-β	Insulin receptor beta 4b8	Cell Signaling, 3025	Rabbit, monoclonal	1:1000
IGF-1Rβ	IGF-1 receptor beta, d23h3	Cell Signaling, 9750	Rabbit, monoclonal	1:1000
β-actin	β-actin (13E5)	Cell Signaling, 4970	Rabbit, monoclonal	1:20 000
Phospho-Akt (Ser473)	Phospho-Akt (Ser473) (D9E) XP	Cell Signaling, 4060	Rabbit, monoclonal	1:2000
Akt	Akt (pan)(11E7)	Cell Signaling, 4685	Rabbit, monoclonal	1:1000

### Quantification of hybrid receptors

Hybrid receptor expression was studied by immunoprecipitation and Western blot analysis. *Immunoprecipitation*: total protein was combined with 30 µL of protein G agarose beads (Roche Diagnostic, Switzerland), 300 µL buffer (100 mM HEPES, pH 7.8, 100 mM NaCl, 10 mM MgSO4, 0.02% Tween-20) and 1:100 dilution of IGF-1 receptor antibody (D23H3). Ag–Ab immune complexes were allowed to form over 3 h at 4°C, after which they were collected using brief centrifugation. Precipitates were washed gently three times in phosphate-buffered saline (PBS)–0.02% Tween-20 before elution with sodium dodecyl sulphate (SDS) buffer. *Western blotting*: Samples were resolved by sodium dodecyl sulfate polyacrylamide gel electrophoresis (SDS-PAGE) and transferred to nitrocellulose membranes. Membranes were probed with IR-β antibody; 1:1000 (4b8), followed by appropriate secondary HRP-conjugated antibody to visualise IR/IGF-1R hybrids. Membranes were re-probed with IGF-1R-β antibody to allow hybrid receptors to be reported as relative levels compared with total IGF-1R protein.

### IR and IGF-1R gene expression

Quantitative real-time polymerase chain reaction (PCR) was used to measure mRNA levels of IR and IGF-1Rs. mRNA from aorta was isolated and purified using the RNeasy mini kit (QIAGEN, Dusseldorf, Germany) as per the manufacturer’s protocol. Reverse transcription was performed using iScript cDNA synthesis kit (Bio-Rad, Hertfordshire, UK). Quantitative PCR was then used to determine IR and IGF-1R mRNA expression using specific TaqMan assays (Invitrogen, IR; Mm01211875_m1, IGF-1R; Mm00802831_m1). Receptor expression was calculated relative to the average of two housekeeping genes – TATA box–binding protein (TBP; Mm01277042_m1) and CyclinB (Mm03053893_gH) – using the formula 2^−ΔCt^.

### Insulin and IGF-1 stimulated Akt phosphorylation in vivo

Mice were injected subcutaneously with either vehicle, native human insulin (Novo Nordisk, Malov, Denmark) or recombinant human IGF-1 (Ipsen, Slough, UK). Dosage was calculated based on the average weight of all lean mice (assuming blood volume does not significantly alter in obese mice). Plasma levels of human IGF-1 and human insulin in the mice were measured using ELISAs (*insulin*; Novo Nordisk, Malov, Denmark. *IGF-1*; Immunodiagnostic Systems, Tyne & Wear, UK) as described previously,^14^ in order to confirm equivalent dosing levels between HF and LF mice. After 15 min stimulation, mice were euthanised and the aorta rapidly harvested and snap-frozen. Twenty micrograms of protein was processed for Western blotting. Nitrocellulose membranes were probed with antibodies diluted in 5% BSA; 1:1000 Akt, 1:2000 phosphorylated Akt (Ser473) and 1:20,000 beta actin (Cell Signaling).

### In vitro assessment of receptor and hybrid expression in human umbilical vein endothelial cells

Cryopreserved human umbilical vein endothelial cells (HUVECs) were purchased from Promocell (Stourbridge, UK) and maintained in culture in endothelial cell growth medium at 37°C in a humidified atmosphere with 5% CO_2_. At ~70% confluency, cells were treated with the following: 100 nM human recombinant insulin (Sigma-Aldrich, Dorset, UK), 100 nM human recombinant IGF-1 (GroPep, Adelaide, Australia), 10 ng/mL TNF-α (PeproTech, London, UK) 1 µM angiotensin II (Sigma-Aldrich, Dorset, UK), 50 µM hydrogen peroxide (H_2_O_2_; Sigma-Aldrich, Dorset, UK), 25 mM glucose and/or 10 nM insulin (Sigma-Aldrich, Dorset, UK) for 24 h in low-serum (0.5%) medium. Whole-cell lysates were prepared in cell extraction buffer and samples processed for Western blot analysis of IR, IGF-1R and hybrid receptors.

### Resistance vessel vasomotor function in response to insulin and IGF-1

Two-millimetre segments of first-order mesenteric arteries were harvested from LT HF and LF mice and mounted in a wire myograph (Danish Myo Technology A/S, Aarhus, Denmark) containing physiological buffer (mM): KCl 7.4, NaCl 118, NaHCO_3_ 15, KH_2_PO_4_ 1.2, MgSO_4_ 1.2, glucose 11, CaCl_2_ 2.5, EDTA 0.023 at 37°C, 5% CO_2_ and 95% O_2_. Vessels were equilibrated at a resting lumen diameter of 0.9 × L100 (L100 represents vessel diameter under passive transmural pressure of 100 mmHg) in buffer for 30 min. Three potassium-induced constrictions were performed using high potassium buffer and vessels constricting less than 1 mN were excluded from the study. Vessels were pre-constricted with phenylephrine, at a dose yielding approximately 40% constriction obtained with high potassium buffer, and left to stabilise for 10 min. Relaxation to cumulative addition of either insulin (0.001 nM/1 pM to 1 µM) or IGF-1 (0.001 nM to 10 nM) was assessed in pre-constricted vessels. A time-matched control recording was also performed following the same protocol, without the addition of insulin or IGF-1. The contractile force of a vessel segment was recorded using PowerLab 4/25–LabChart7 acquisition system (ADInstruments, Oxford, UK).

### Ex vivo analysis of insulin and IGF- 1 induced Akt phosphorylation in resistance arteries

First-order mesenteric artery segments of 5 mm length were placed into Krebs Ringer solution and stimulated with insulin or IGF-1 at different concentrations (0.001 nM to 1 µM) for 15 min at 37°C. Stimulated vessels were snap-frozen, then lysed and sonicated. Samples were analysed by SDS-PAGE and Western blotting.

### Statistical methods

Data were analysed using GraphPad Prism software (version 7). For animal studies, one-way analysis of variance (ANOVA) was used to compare the mean value across groups, followed by Tukey’s multiple comparisons test. Where differences between two groups were analysed, an unpaired t-test was used with Welch’s correction. To study differences in vitro, a paired two-way t-test was utilised.

The results are given as mean ± standard error of the mean (SEM). In this study, differences with a *p* value of <0.05 were considered statistically significant. *Multivariate and univariate analysis*: Uni- and multivariate linear regression analysis was performed using SPSS version 21 (IBM Corporation, Armonk, NY) to determine the association between receptor abundance and selected covariates. Standardised regression (beta) coefficients are presented, with * denoting statistical significance at *p* < 0.05.

## Results

### Progressive decline in insulin and IGF-1 sensitivity in obesity

We fed mice a HF, obesogenic diet for 2, 5 or 16 weeks; this led to progressive metabolic impairment in comparison to LF fed controls (summarised in Table 2).

**Table 2.** Metabolic effects of high-fat (HF) calorie diet for 2, 5 and 16 weeks.

	2 weeks		5 weeks		16 weeks	
	LF	HF	LF	HF	LF	HF
Weight (g)	26.0 ± 0.4	29.2 ± 0.6^a^	25.4 ± 0.5	30.8 ± 0.5^a^	29.8 ± 0.4	50.6 ± 0.6^a^
Fasting glucose (mmol/L)	7.7 ± 0.3	9.7 ± 0.3^b^	9.3 ± 0.3	13.1 ± 0.3^a^	8.6 ± 0.2	12.7 ± 0.5^a^
GTT-AUC (mmol/L × time)	19.8 ± 1.1	38.3 ± 2.0^a^	11.2 ± 1.2	23.6 ± 1.5^a^	10.1 ± 0.8	46.7 ± 3.1^a^
Plasma insulin (ng/mL)	0.7 ± 0.2	1.8 ± 0.5^c^	0.6 ± 0.1	1.8 ± 0.2^a^	0.8 ± 0.06	5.9 ± 0.4^a^
Plasma IGF-1 (ng/mL)	273 ± 21.9	281.9 ± 33.6	230 ± 7.7	277 ± 10.6^b^	219 ± 14	408 ± 21.6^a^

‘GTT-AUC denotes GTT-Area Under Curve LF’ denotes lean diet, *n* = 10–25 in each group.

Data are reported as means ± SEM.

a *p* < 0.0001.

b *p* < 0.001.

c *p* < 0.05 compared to the respective control (LF) group.

### IGF-1R, IR and IGF-1R/IR hybrid receptor expression in aorta during obesity

**Figure 1.**
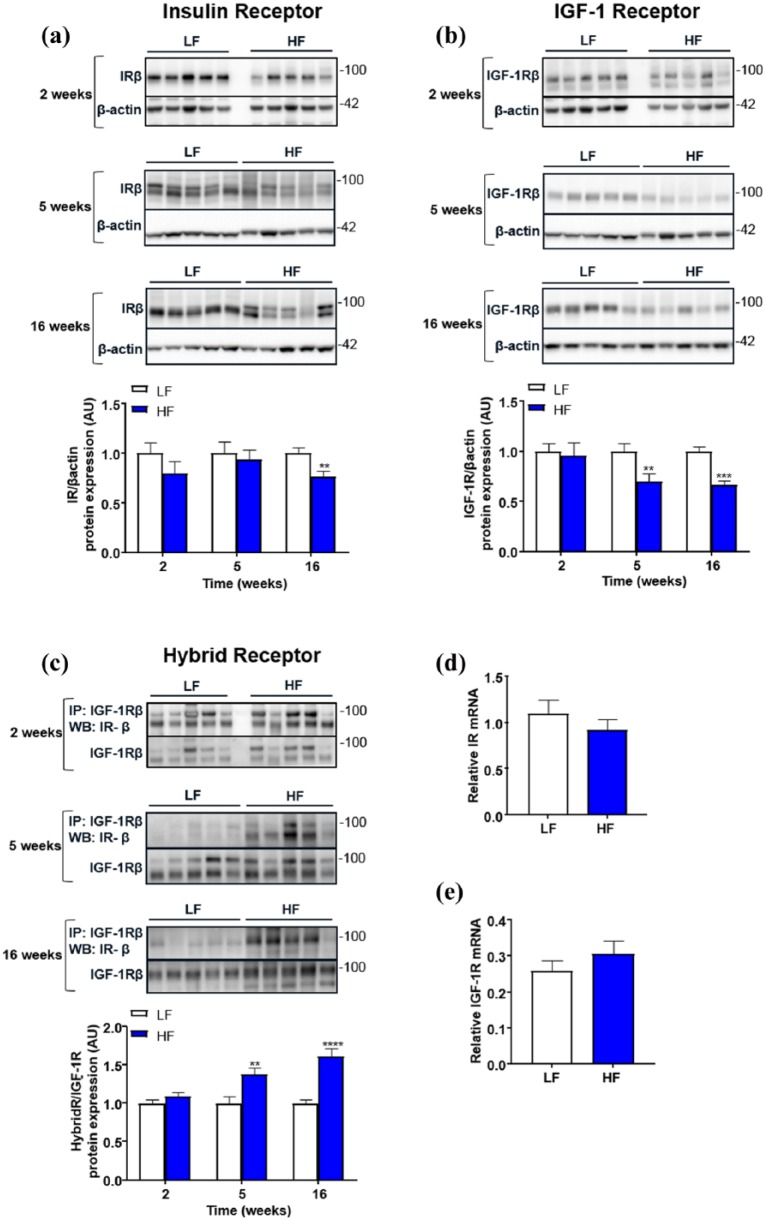
Temporal effects of obesity on IR, IGF-1R and hybrid receptor expression in aorta of low-fat (LF) and high-fat (HF) diet fed mice. Data show changes in (a) IR, (b) IGF-1R and (c) hybrid receptor protein at 2, 5 and 16 weeks of feeding. Representative Western blot images are shown with densitometry (a, b and c, *n* = 10–25 in each group). Relative (d) IR and (e) IGF-1R mRNA is shown in LF and HF mouse aortae at 16 weeks feeding (*n* = 6 in each group). Analysis was performed between gels, and samples were normalised to a single control whole cell lysate which was loaded on all gels. All data are given as mean values ± SEM. **p < 0.01, ***p < 0.001, ****p < 0.0001 versus lean group.

We studied changes in IR, IGF-1R and hybrid receptor expression in aortic lysates from mice after 2, 5 and 16 weeks of HF and LF. In tissue samples, IR was observed as a double band migrating at 80–100 kDa. We observed IR as a single or double band in tissue but not cell lysates. We suggest this is due to the varying degrees of IR glycosylation in different cell types and tissues, resulting in two migrating populations of IR. We did not observe any discernible difference in the two populations of IR when comparing HF and LF. The level of hybrid receptors was studied by immunoprecipitating IGF-1R and detecting IR in the hybrid receptor by Western blot. The relative level of IR compared to total IGF-1R was determined. The effect of an obesogenic diet was studied over time. After 2 weeks feeding, IR, IGF-1R and hybrid receptor protein expression was unchanged (Figure 1). After 5 weeks of HF, IR expression in aorta was unchanged (Figure 1(a)), whereas IGF-1R expression had declined by 30% (Figure 1(b)) and hybrid receptor expression increased by 38% (Figure 1(c)). After 16 weeks of HF, IR expression had declined by 24% (Figure 1(a)), IGF-1R expression had declined further by 34% (Figure 1(b)) and hybrid receptor expression increased by 62% (Figure 1(c)).

To determine whether the reduction in receptor expression was due to transcriptional changes, real-time PCR was performed on RNA isolated from aorta of mice after 16 weeks of feeding. No changes were observed in IR (Figure 1(d)) or IGF-1R (Figure 1(e)) relative mRNA expression between HF and LF fed mice.

Univariate correlates of hybrid receptor expression were plasma insulin, plasma IGF-1, fasting glucose, body weight, dietary fat content and duration of diet, all *p* < 0.05. In multivariate analysis, independent predictors of hybrid expression were dietary fat content, duration of diet ingestion and plasma IGF-1, all *p* < 0.05 (Table 3).

**Table 3.** Independent predictors of hybrid receptor (HR), insulin receptor (IR) and IGF-1 receptor (IGF-1R) expression in mouse aorta.

Covariate	Univariate correlation with			Multivariate correlation with^a^		
	HR	IR	IGF-1R	HR	IR	IGF-1R
Dietary fat (%)	0.502*	−0.119	−0.161	0.325*	−0.002	−0.007
Diet duration (weeks)	0.348*	−0.386*	−0.556*	0.304*	−0.411*	−0.581*
Body mass (g)	0.585*	−0.168	−0.315*	−0.231	0.185	0.225
Capillary glucose (mmol/L)	0.319*	−0.538*	−0.573*	−0.035	−0.654*	−0.61*
Plasma insulin	0.572*	−0.137	−0.242*	0.282	−0.18	−0.136
Plasma IGF-1	0.536*	0.17	−0.024	0.276*	0.516*	0.3*

IGF-1: insulin-like growth factor-1.

β coefficients presented.

a Presented multivariate correlation coefficients account for all five other listed covariates.

* *p* < 0.05.

### IGF-1R, IR and IGF-1R/IR hybrid expression in vitro in response to different components of the obesity phenotype

**Figure 2.**
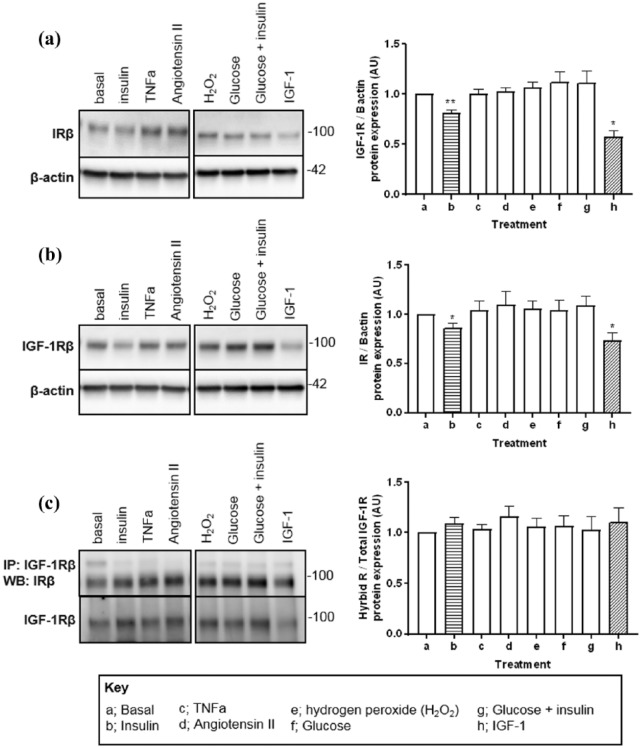
Effects of supplementation of obesity-related modulators on receptor expression in human umbilical vein endothelial cells in vitro. Data show effects of physiological modulators on (a) IR, (b) IGF-1R and (c) hybrid receptor protein expression. Representative Western blot images are shown with densitometry. All data are given as mean values ± SEM. a: basal (0.5% low-serum medium); b: insulin (100 nM); c: TNF-α (10 ng/mL); d: angiotensin 2 (1 µM); e: H_2_O_2_ (50 µM); f: glucose (25 mM); g: glucose (25 mM) + insulin (10 nM); h: IGF-1 (100 nM). *p < 0.05, **p < 0.01 versus control (basal) group (*n*  = 6 for each).

We cultured HUVECs in conditions aiming to recapitulate different components of the obesity phenotype including elevated: insulin, IGF-1, glucose with and without insulin, angiotensin II, hydrogen peroxide and TNF-α for 24 h. Only insulin and IGF-1 reduced expression of their respective receptors despite this hybrid receptor expression remained unchanged (Figure 2).

### Temporal effects of obesity on IGF-1 and insulin stimulated Akt phosphorylation

To examine the temporal effect of obesity on insulin and IGF-1-mediated phosphorylation of the key signalling kinase Akt, we performed in vivo administration of either insulin or IGF-1 to HF and LF fed mice after 2, 5 and 16 weeks.

**Figure 3.**
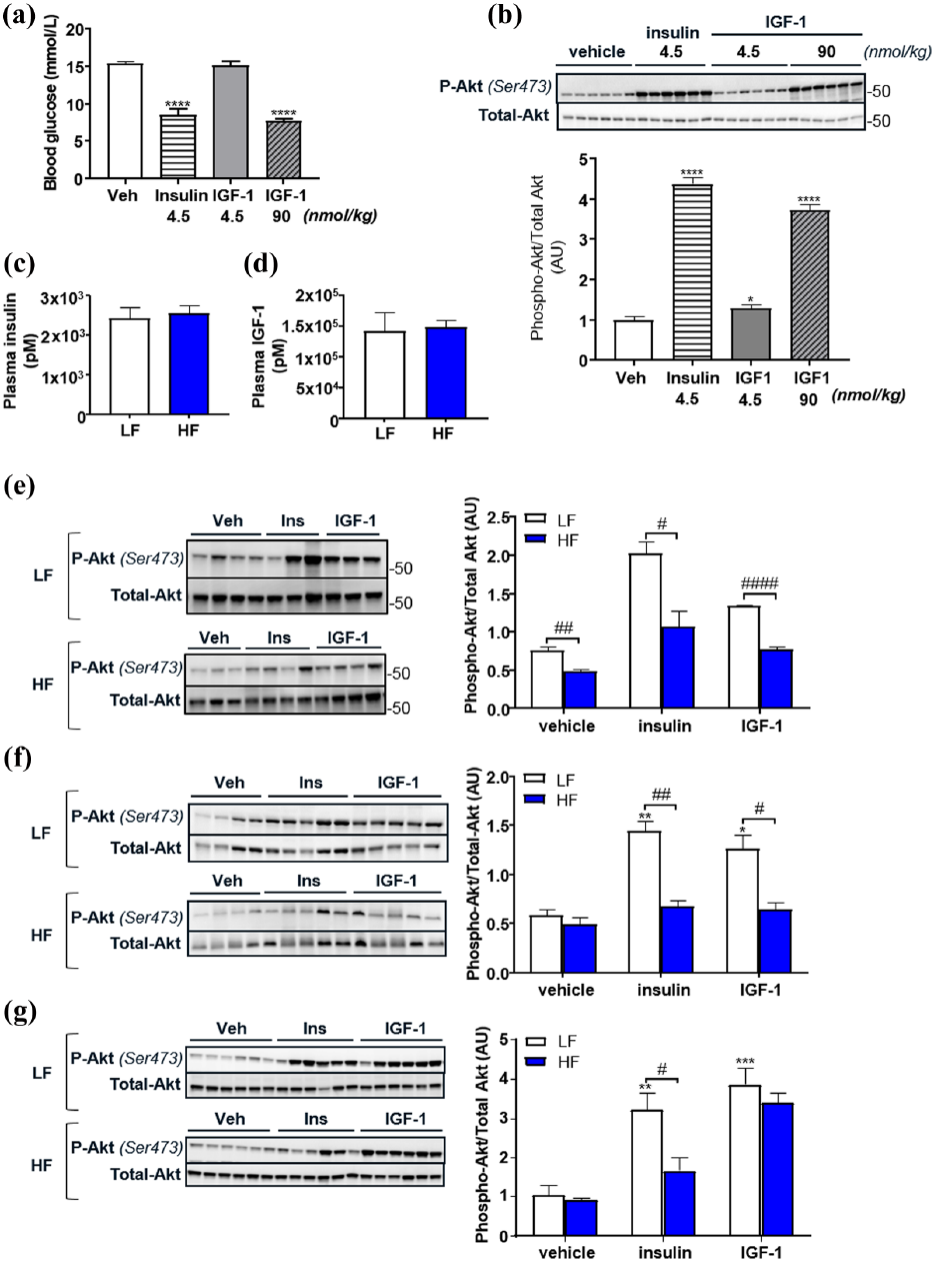
Temporal effects of high-fat (HF) diet-induced obesity on insulin and IGF-1 stimulated Akt phosphorylation compared to lean low-fat (LF) diet fed mice. Reduction in blood glucose is shown in response to insulin (4.5 nmol/kg) and IGF-1 at equimolar (4.5 nmol/kg) and equipotent (90 nmol/kg) doses in lean mice (a). Phosphorylation of Akt in the aorta of lean mice in response to insulin (4.5 nmol/kg) and equipotent and equimolar doses of IGF-1 (b). Data show level of subcutaneously injected human insulin (c) and IGF-1 (d) in plasma of LF and HF diet fed mice. Differences in insulin (4.5 nmol/kg) and IGF-1 (90 nmol/kg) stimulated phosphorylation of Akt in LF and HF mouse aortae are shown at 2 weeks (e), 5 weeks (f) and 16 weeks (g) of feeding. Representative Western blots and densitometry are shown. All data are given as mean values ± SEM (*n* = 6–8 for each group). Bars represent comparisons made between HF and lean groups. *p < 0.05, **p < 0.01, ***p < 0.001, ****p < 0.0001 versus lean vehicle group.

To determine the optimum dose of IGF-1, we first performed a study in lean mice to examine the effect of equimolar and equipotent (as determined by blood glucose lowering ability) doses of IGF-1 and insulin on aortic Akt phosphorylation. When equimolar concentrations of insulin (4.5 nmol/kg) or IGF-1 (4.5 nmol/kg) were administered, insulin led to a greater decrement in blood glucose and greater increment in phosphorylation of Akt in aorta than IGF-1 (Figure 3(a) and (b)). An IGF-1 dose of 90 nmol/kg stimulated similar blood glucose lowering and Akt phosphorylation as 4.5 nmol/kg insulin. Therefore, in subsequent studies, we used equipotent doses; insulin at 4.5 nmol/kg and IGF-1 at 90 nmol/kg. To ensure that plasma exposure levels would be comparable between LF and HF mice, doses for all mice were calculated based on the average body weight of the LF mice. Plasma exposure levels of human insulin and IGF-1 was assessed with specific ELISAs for insulin and IGF-1 and we found comparable levels between the LF and HF groups.

After 2 weeks HF, despite no change in receptor expression, both insulin and IGF-1-mediated Akt phosphorylation were blunted (Figure 3(e)). After 5 weeks, HF both insulin and IGF-1-mediated Akt phosphorylation were blunted (Figure 3(f)). By 16 weeks, however, while insulin-mediated Akt phosphorylation remained blunted, IGF-1 mediated Akt phosphorylation was similar in LF and HF fed mice (Figure 3(g)), possibly reflecting an increase in hybrid receptor expression.

### Resistance vessel relaxation and Akt phosphorylation in response to insulin and IGF-1

**Figure 4.**
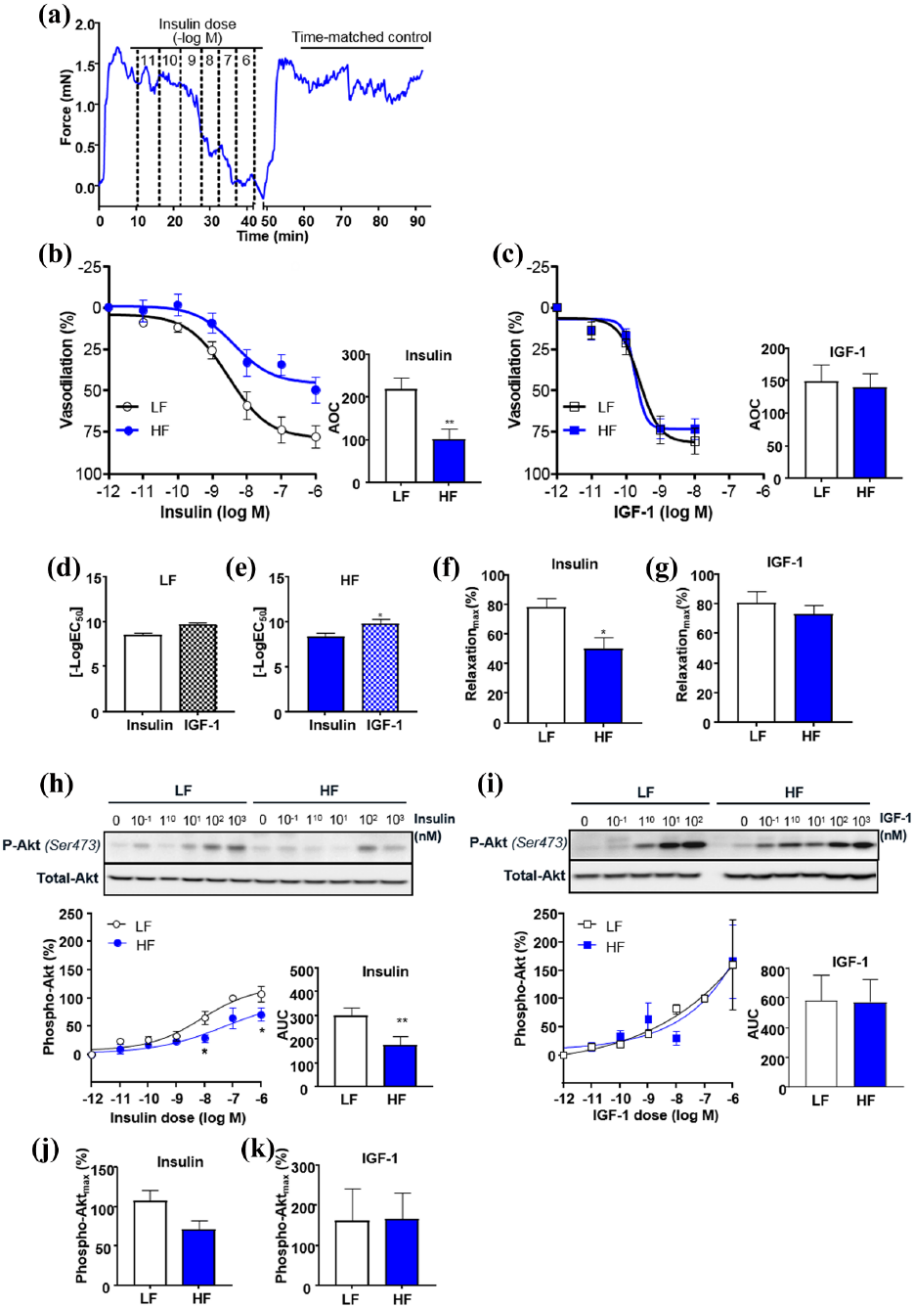
Effects of high fat (HF) diet–induced obesity on mesenteric artery function in response to insulin and IGF-1 compared to lean low fat (LF) diet fed mice. (a) Data show a representative recording of dose-dependent insulin-induced vasorelaxation followed by a time-matched control showing stability of pre-constriction over time. Data show (b) insulin-induced and (c) IGF-1-induced relaxation in pre-constricted mesenteric arteries (first order) taken from LF and HF mice after 16 weeks feeding. Differences in vascular sensitivity to insulin and IGF-1 in (d) LF and (e) HF mice are shown and maximal relaxation achieved with (f) insulin and (g) IGF-1. (h) Insulin and (i) IGF-1-mediated phosphorylation of Akt in LF and HF mesenteric arteries is shown with maximal phosphorylation shown in (i) and (k). All data are given as mean values ± SEM. *p < 0.05, HF versus LF group (*n* = 3–7 for each group).

We previously demonstrated that 8 weeks HF led to blunting of both insulin and IGF-1-mediated vasorelaxation of the aorta;^2^ however, this study did not examine the effect of obesity on resistance vessel function. Here, we show that both insulin and IGF-1 led to vasorelaxation of first-order mesenteric arteries (Figure 4(a) to (c)). IGF-1, however, was more potent than insulin (Figure 4(d) and (e)). HF resulted in blunted insulin-mediated vasorelaxation (Figure 4(f)) but IGF-1-mediated responses were unaffected (Figure 4(g)). A dose-dependent increase in phosphorylation of Akt was observed with increasing concentrations of insulin and IGF-1 (Figure 4(h) and (i)); however, IGF-1 treatment led to a greater maximal response (Figure 4(j) and (k)). HF-blunted insulin-mediated Akt phosphorylation in first-order mesenteric arteries (Figure 4(h)), but IGF-1-mediated Akt phosphorylation was unaffected by HF (Figure 4(i)).

## Discussion

This report describes a number of novel findings of relevance to our understanding of obesity, metabolic disease and the insulin/IGF-1 system, including (1) IR/IGF-1R hybrid protein level in aorta does not appear to be a primary function of mRNA IR and IGF-1R levels; (2) independent in vivo correlates of hybrid expression are plasma IGF-1, HF and duration of HF feeding period; (3) IGF-1 is a more potent activator of Akt and vasorelaxation in resistance vessels than insulin, whereas we have shown previously that the opposite is true in larger conduit vessels; and (4) after 16 weeks of HF diet, IGF-1-mediated responses in resistance vessels and conduit vessels are preserved, whereas insulin-induced responses are blunted.

### Hybrid receptor expression does not appear to be simply dependent on the molar fraction of each receptor

Energy and nutrient homeostasis in mammals requires tight regulation and integration of multiple systems, which during periods of cellular and whole organism stress, couple nutrient delivery to energy storage, cell growth and tissue repair.^15^ Integral to nutrient homeostasis is the insulin/IGF-1 system, the development and evolution of which occurred before the relatively unusual environmental circumstances of caloric excess experienced by 21st-century humans.^16^ As a result, the insulin/IGF-1 system is unable to effectively adapt to the challenge posed by chronic calorie excess and gradually deteriorates giving rise to insulin-resistant type 2 diabetes mellitus and its lethal complications, many of which involve the cardiovascular system.^17^

A hallmark of type 2 diabetes is the increased expression of IR/IGF-1R hybrids which are thought to restrict insulin signalling in favour of IGF-1,^5^ a scenario we,^18,19^ and others, have demonstrated may be present in the endothelium^20^ and vasculature.^21^ Understanding how hybrid receptors are regulated and activated in the vasculature is hence of importance to our understanding of obesity-related perturbation of insulin signalling and vascular dysfunction. In this study, and consistent with cross-sectional studies in humans,^10^ increased hybrid receptor expression was preceded by insulin (and IGF-1) resistance. We also show that expression of hybrid receptors is closely linked to the duration of high-fat diet ingestion and plasma IGF-1 level. In contrast to elegant studies from Federici et al.,^22^ we did not demonstrate independent correlations between hybrid receptor expression and blood glucose or insulin concentration,^23^ rather IGF-1 concentration more closely correlated with hybrid expression. It is possible that this is due to the presence of obesity rather than the primary hyperinsulinaemia described by Federici et al.^23^ An additional explanation could be the use of different tissues – muscle samples as studied by Federici et al may show more sensitivity to hybrid formation following perturbations in insulin and glucose, whereas vascular tissue as in this study may be more sensitive to changes in IGF-1 levels. After 16 weeks of HF, we observed no change in receptor mRNA levels yet found that IR and IGF-1R protein decreased in obese mice. Despite this reduction in total receptor level, the relative expression of hybrid receptors increased. This suggests that regulation of IR and IGF-1R occurs at the translational level or it could be speculated that the internalisation/degradation pathways of the receptors are distinct from hybrid receptors and they are more readily influenced by hormone exposure levels.

### Insulin and IGF-1 in resistance vessel function

We previously showed that obesity leads to resistance to both IGF-1 and insulin-mediated activation of eNOS and relaxation of the aorta.^2^ Studies in humans have shown that IGF-1 increases forearm blood flow consistent with an effect on resistance vessels.^24^ McCallum et al.^25^ showed that IGF-1-mediated vasodilatation of aorta is blunted in hypertensive rats and Hasdai et al.^26^ showed that arteriolar vasorelaxation to IGF-1 is attenuated in experimental hypercholesterolaemia. The effect of obesity on insulin and IGF-1-mediated responses in resistance vessels has been unclear. Here, we show that IGF-1 relaxes resistance vessels and is more potent than insulin. We also show the intriguing finding that obesity blunts insulin-mediated resistance vessel relaxation and Akt phosphorylation, while IGF-1-mediated vasorelaxation and Akt phosphorylation remained intact. These findings reveal a potentially important divergence between insulin and IGF-1 responses in resistance vessels with preservation of IGF-1 responses, when we previously showed that obesity leads to IGF-1 resistance in aorta.^2^

### Study limitations

A number of limitations should be discussed: we used the semi-quantitative approach of expression levels of receptors to estimate receptor numbers so we cannot comment on the exact numerical relationship between IR and IGF-1R in relation to hybrid receptor formation. In resistance vessels, we were unable to quantify receptor expression due to limited amounts of protein available; it would be of interest in the future to examine receptor expression in resistance vessels as obesity progresses.

## Conclusion

We have provided a number of insights into changes in the IR/IGF-1R/hybrid receptor system as obesity progresses, showing that after long-term obesity, IGF-1-mediated Akt phosphorylation is preserved in aorta and resistance vessels. Moreover, we show that IGF-1 is a more potent vasodilator of resistance vessels than insulin, and after 16 weeks of high-fat diet, while insulin-mediated resistance vessel function is blunted, IGF-1 responses are maintained. These data raise the intriguing possibility that using IGF-1 or manipulating hybrid expression may be an approach to treat obesity-related vascular dysfunction, a possibility that warrants future work.
